# Design, synthesis and anticancer activity of some novel thioureido-benzenesulfonamides incorporated biologically active moieties

**DOI:** 10.1186/s13065-016-0161-4

**Published:** 2016-04-07

**Authors:** Mostafa M. Ghorab, Mansour S. Alsaid, Mohamed S. Al-Dosary, Yassin M. Nissan, Sabry M. Attia

**Affiliations:** Department of Pharmacognosy, College of Pharmacy, King Saud University, P.O. Box 2457, Riyadh, 11451 Saudi Arabia; Department of Drug Radiation Research, National Center for Radiation Research & Technology, Atomic Energy Authority, Cairo, Egypt; Department of Pharmaceutical Chemistry, Faculty of Pharmacy, Cairo University, Cairo, Egypt; Department of Pharmacology and Toxicology, College of Pharmacy, King Saud University, P.O. Box 2457, Riyadh, 11451 Saudi Arabia

**Keywords:** Synthesis, Sulfonamides, Thioureido, Anticancer activities

## Abstract

**Background:**

Many thiourea derivatives have exhibited biological activities including anticancer activity through several mechanisms. On the other hand, benzenesulfonamide derivatives have proven to be good anticancer agents. Hybrids of both moieties could be further developed to explore their biological activity as anticancer.

**Results:**

Novel series of thioureidobenzenesulfonamides incorporating miscellaneous biologically active moieties **3**–**17** were designed and synthesized utilizing 4-isothiocyanatobenzenesulfonamide **2** as strategic starting material. The structures of the newly synthesized compounds were established on the basis of elemental analyses, IR, ^1^H-NMR, ^13^C-NMR and mass spectral data. All the newly synthesized compounds were evaluated for their in vitro anticancer activity against various cancer cell lines. Most of the synthesized compounds showed good activity, especially compounds **3**, **6**, **8**, **9**, **10**, **15** and **16** which exhibited good activity higher than or comparable to the reference drugs, DCF and Doxorubicin, except breast cancer line. As a trial to suggest the mechanism of action of the active compounds, molecular docking on the active site of mitogen kinase enzyme (MK-2) was performed and good results were obtained especially for compound **3**.

**Conclusion:**

Compounds **3**, **6**, **8**, **9**, **10**, **15** and **16** may represent good candidates for further biological investigations as anticancer agents. Their cytotoxic activity could be due to their action as MK-2 enzyme inhibitors.

**Graphical abstract:**
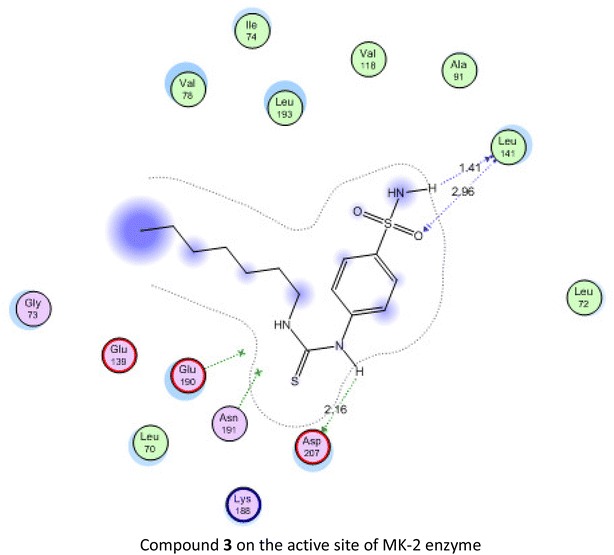
Compound **3** on the active site of MK-2 enzyme

## Background

Various types of cancer are now considered as the second cause of death after cardiovascular disorders [1]. The inability of the present anticancer chemotherapeutic agents to discriminate between normal cells and cancer cells comprises the biggest challenge for successful cancer treatment [2]. Serious side effects of anticancer chemotherapeutic agents limit their usage and in many cases surgery or radiotherapy replace them [3]. The continuous seek for safer and more effective anticancer agents is still a major goal for medicinal chemists.

Thiourea is a versatile synthetic block for the synthesis of a wide variety of new organic compounds with biological activity including antimicrobial, antifungal, antidiabetic, antimalarial, anti HIV and CNS active drugs [4–11]. Many of aryl thiourea derivatives have applications in medicine, industry and agriculture [12–15]. Thiourea was incorporated in many tyrosine kinase inhibitors because of its ability to form powerful hydrogen bonds in the ATP binding pocket of the enzymes [16]. The thiourea derivative YH345 **A** has shown strong protein farnesyl transferase inhibition activity [17]. Also several heterocyclic thiourea derivatives have shown strong DNA topoisomerase inhibitory activity [18].

On the other hand, sulfonamide derivatives posses a wide range of biological activity including antibacterial, anticonvulsant, anti-inflammatory and anticancer activity [19–22]. The mechanism of anticancer activity may involve a wide range of different mechanisms, such as cell cycle arrest in the G1 phase [23] and inhibition of carbonic anhydrase [24], histone deacetylases (HDACs) [25], methionine amino peptidases (MetAPs) [26], matrix metalloproteinase (MMPs) [27], nicotinamide adenine dinucleotide (NADH) oxidase [28], cyclin-dependentkinase (CDK) [29], binding to β-Tubulin, and disruption of microtubule assembly [30]. Indisulam (E7070) **B** is an example of an anticancer agent that contains sulfonamide moiety [31].

Based on the previous facts and as a continuation of our previous work in the seek of novel anticancer agents [32–38], we herein report the synthesis and biological evaluation of new sulfonamide thiourea derivatives **3**–**17** presented by general structure **C** as hybrid molecules of benzensulfonamide and thiourea moieties as anticancer agents. Molecular docking of the active newly synthesized compounds was performed on the active site of mitogen activated kinase enzyme (MK-2) in a trial to suggest a mechanism of action for their cytotoxic activity.

## Results and discussion

### Chemistry

**Scheme 1:**
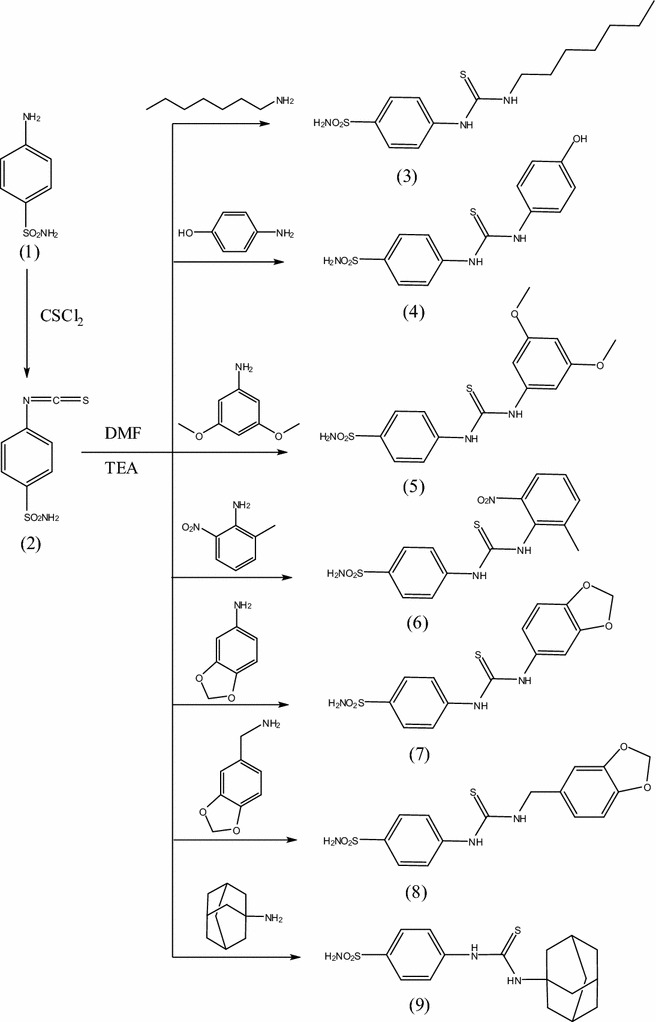
Synthesis of compounds **1**–**9**

**Scheme 2:**
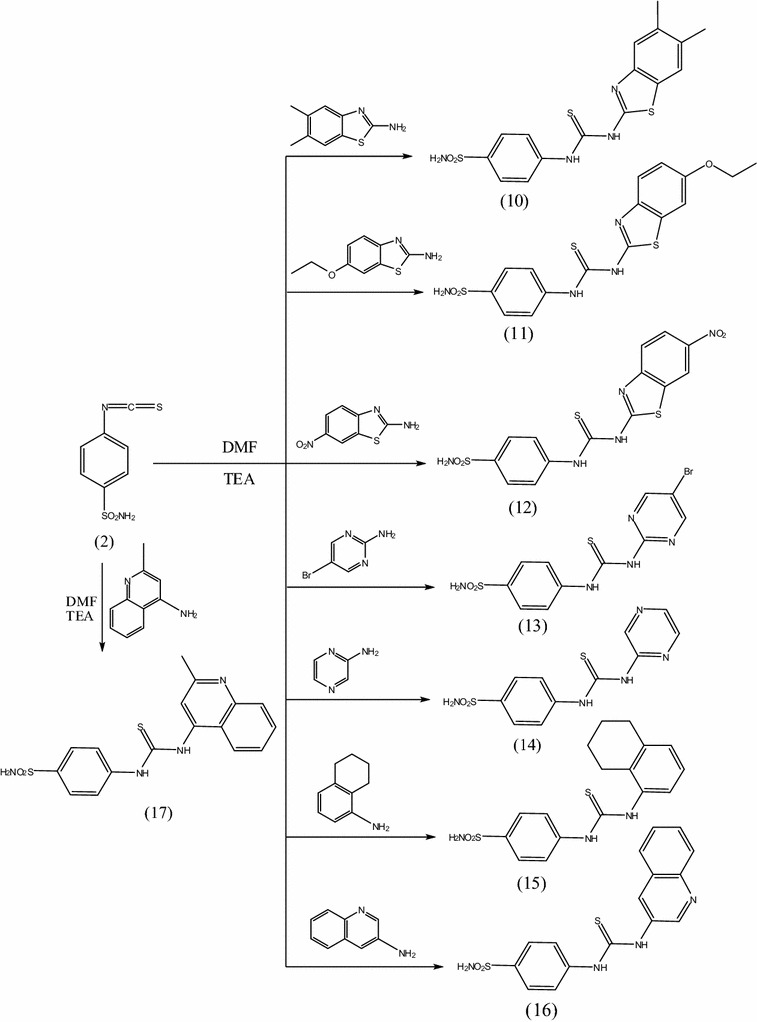
Synthesis of compounds **10**–**17**

The aim of this work was to design and synthesize a new series of thioureidobenzenesulfonamide derivatives having miscellaneous biologically active moieties to evaluate their anticancer activity. Thus, interaction of 4-isothiocyanatobenzenesulfonamide **2** with several amines in dry *N*,*N*-dimethylformamide containing triethlyamine as catalyst afforded the corresponding thioureidobenzenesulfonamude derivatives **3**–**17** (Schemes 1 and 2). The structures of the obtained compounds were established on the basis of elemental analyses and spectral data.IR spectra of compounds **3**–**17** showed the absence of N=C=S group and presence absorption bands for (NH), (CH arom.), (CH aliph.), (C=S) and (SO_2_). ^1^H-NMR spectra of compounds **3**–**17** exhibited a singlets at 7.8–13.8 ppm assigned to 2NH groups of thiourea which were exchanged upon duetration.IR spectrum of compound **3** showed the characteristic bands at 3312, 3214 cm^−1^ (NH), 3099 cm^−1^ (CH arom.), 2202 cm^−1^ (C≡N), 1655 cm^−1^ (C=O), 1387, 1157 cm^−1^ (SO_2_), 1250 cm^−1^ (C=S). ^1^H-NMR spectrum of compound **3** exhibited a triplet signal at 0.8 ppm due to CH_3_, a multiplet at 1.2–1.4 ppm due to 5CH_2_, a mutiplet at 3.3 ppm due to NHCH_2_ and singlets at 9.3 and 10.4 ppm assigned to 2NH groups which were exchangeable with D_2_O. Mass spectrum of compound **3** revealed a molecular ion peak m/z at of 329 (M^+^) (14.41) with a base peak appeared at 155 (100). ^13^C-NMR spectrum of compound **3** exhibited signals at 177.4 ppm assigned to (C=S). IR spectrum of compound **4** showed the characteristic bands at 3363 cm^−1^ (OH), 3280, 3143 cm^−1^ (NH, NH_2_), 3090 cm^−1^ (CH arom.), 1393, 1182 cm^−1^ (SO_2_), 1274 cm^−1^ (C=S). ^1^H-NMR spectrum of compound **4** exhibited signals at 10.2, 11.4 attributed to 2NH groups and 13.1 ppm assigned to OH group which exchangeable with D_2_O. Mass spectrum of compound **4** revealed a molecular ion peak m/z at of 323 (M^+^) (9.03) with a base peak appeared at 91 (100). ^13^C-NMR spectrum of compound **4** showed signals at 180.1 ppm assigned to (C=S).IR spectrum of compound **5** revealed the characteristic bands at 3317, 3254, 3173 cm^−1^ (NH, NH_2_), 3100 cm^−1^ (CH arom.), 2963, 2938, 2829 cm^−1^ (CH aliph.), 1363, 1156 cm^−1^ (SO_2_), 1259 cm^−1^ (C=S). ^1^H-NMR spectrum of compound **5** exhibited singlet at 3.9 ppm attributed to 3OCH_3_ groups and singlet at 9.8 ppm assigned to 2NH groups, which exchangeable with D_2_O. Mass spectrum of compound **5** revealed a molecular ion peak m/z at of 367 (M^+^) (17.8) with a base peak appeared at 76 (100). ^13^C-NMR spectrum of compound **5** exhibited signals at 179.3 ppm assigned to (C=S).IR spectrum of compound **6** showed the characteristic bands at 3353, 3243, 3171 cm^−1^ (NH, NH_2_), 3009 cm^−1^ (CH arom.), 1340, 1161 cm^−1^ (SO_2_), 1290 cm^−1^ (C=S). ^1^H-NMR spectrum of compound **6** revealed singlet at 10.3 ppm assigned to 2NH groups, which exchangeable with D_2_O. Mass spectrum of compound **6** exhibited a molecular ion peak m/z at of 366 (M^+^) (15.8) with a base peak appeared at 133 (100). ^13^C-NMR spectrum of compound **6** exhibited singlet at 180.1 ppm assigned to (C=S). IR spectrum of compound **7** exhibited the characteristic bands at 3325, 3241 cm^−1^ (NH, NH_2_), 3100 cm^−1^ (CH arom.), 1331, 1156 cm^−1^ (SO_2_), 1241 cm^−1^ (C=S). ^1^H-NMR spectrum of compound **7** showed singlet at 9.5 ppm assigned to 2NH groups, which exchangeable with D_2_O. Mass spectrum of compound **7** revealed a molecular ion peak m/z at of 351 (M^+^) (34.64) with a base peak appeared at 93 (100). ^13^C-NMR spectrum of compound **7** showed signal at 180.6 ppm assigned to (C=S). IR spectrum of compound **8** exhibited the characteristic bands at 3384, 3348, 3206 cm^−1^ (NH, NH_2_), 3003 cm^−1^ (CH arom.), 1377, 1185 cm^−1^ (SO_2_), 1294 cm^−1^ (C=S). ^1^H-NMR spectrum of compound **8** showed singlet at 7.8 ppm attributed to 2NH groups, which exchangeable with D_2_O. Mass spectrum of compound **8** exhibited a molecular ion peak m/z at of 365 (M^+^) (18.42) with a base peak appeared at 135 (100). ^13^C-NMR spectrum of compound **8** showed signal at 161.1 ppm attributed to (C=S). IR spectrum of compound **9** revealed the characteristic bands at 3434, 3354 cm^−1^ (NH, NH_2_), 3100 cm^−1^ (CH arom.), 2997, 2906, 2851 (CH aliph.), 1396, 1186 (SO_2_), 1282 (C=S). ^1^H-NMR spectrum of compound **9** exhibited multiplet at 1.9 ppm due to 6CH_2_, a multiplet at 2.2–2.4 due to 3CH and singlet at 11.4 ppm due to 2NH groups, which exchangeable with D_2_O. Mass spectrum of compound **9** exhibited a molecular ion peak m/z at of 366 (M^+^) (9.32) with a base peak appeared at 154 (100). ^13^C-NMR spectrum of compound **9** showed singlet at 179.9 ppm due to (C=S). IR spectrum of compound **10** showed the characteristic bands at 3359, 3257, 3143 cm^−1^ (NH, NH_2_), 3031 cm^−1^ (CH arom.), 2954, 2851 cm^−1^ (CH aliph.), 1594 cm^−1^ (C=N), 1381, 1186 cm^−1^ (SO_2_), 1296 cm^−1^ (C=S). ^1^H-NMR spectrum of compound **10** revealed singlets at 10.2 and 13.0 ppm assigned to 2NH groups, which exchangeable with D_2_O. Mass spectrum of compound **10** showed a molecular ion peak m/z at of 393 (M^+^) (16.9) with a base peak appeared at 162 (100). ^13^C-NMR spectrum of compound **10** showed singlet at 180.0 ppm attributed to (C=S). IR spectrum of compound **11** revealed the characteristic bands at 3410, 3334, 3195 (NH, NH_2_), 3069 (CH arom.), 2974, 2925, 2843 (CH aliph.), 1595 (C=N), 1393, 1123 (SO_2_), 1256 (C=S). ^1^H-NMR spectrum of compound **11** revealed a triplet at 1.3 due to CH_3_, a quartet at 4.0 ppm due to CH_2_ and a singlet at 10.3 and 11.2 ppm due to 2NH groups, which exchangeable with D_2_O. Mass spectrum of compound **11** exhibited a molecular ion peak m/z at of 409 (M^+^) (1.85) with a base peak appeared at 156 (100). ^13^C-NMR spectrum of compound **11** revealed singlet at 180.1 ppm assigned to (C=S). IR spectrum of compound **12** revealed the characteristic bands at 3384, 3261, 3165 (NH, NH_2_), 3097 (CH arom.), 1595 (C=N), 1331, 1185 (SO_2_), 1252 (C=S). ^1^H-NMR spectrum of compound **12** exhibited singlet at 10.5 and 12.0 ppm due to 2NH groups, which exchangeable with D_2_O. Mass spectrum of compound **12** exhibited a molecular ion peak m/z at of 409 (M^+^) (13.43) with a base peak appeared at 178 (100). ^13^C-NMR spectrum of compound **12** showed singlet at 179.9 ppm assigned to (C=S). IR spectrum of compound **13** revealed the characteristic bands at 3326, 3175 (NH, NH_2_), 3088 (CH arom.), 1572 (C=N), 1356, 1192 (SO_2_), 1211 (C=S). ^1^H-NMR spectrum of compound **13** exhibited singlet at 12.4 ppm. due to 2NH groups, which exchangeable with D_2_O. Mass spectrum of compound **13** exhibited a molecular ion peak m/z at of 388 (M^+^) (11.81) with a base peak appeared at 157 (100). ^13^C-NMR spectrum of compound **13** showed singlet at 178.6 ppm attributed to (C=S). IR spectrum of compound **14** showed the characteristic bands at 3378, 3240, 3155 (NH, NH_2_), 3100 (CH arom.), 1601 (C=N), 1346, 1199 (SO_2_), 1270 (C=S). ^1^H-NMR spectrum of compound **14** revealed singlets at 11.3, 13.0 ppm attributed to 2NH groups. Mass spectrum of compound **14** showed a molecular ion peak m/z at of 309 (M^+^) (12.83) with a base peak appeared at 79 (100). ^13^C-NMR spectrum of compound **14** showed singlet at 179.0 ppm attributed to (C=S). IR spectrum of compound **15** revealed the characteristic bands at 3413, 3354, 3152 (NH, NH_2_), 3083 (CH arom.), 2982, 2935, 2831 (CH aliph.), 1351, 1159 (SO_2_), 1264 (C=S). ^1^H-NMR spectrum of compound **15** exhibited multiplet at 1.8–2.8 ppm due to 4CH_2_ and singlet at 9.0 ppm due to 2NH groups. Mass spectrum of compound **15** exhibited a molecular ion peak m/z at of 361 (M^+^) (26.34) with a base peak appeared at 177 (100). ^13^C-NMR spectrum of compound **15** showed singlet at 181.5 ppm assigned to (C=S). IR spectrum of compound **16** revealed the characteristic bands at 3373, 3246, 3164 (NH, NH_2_), 3077 (CH arom.), 1595 (C=N), 1365, 1150 (SO_2_), 1293 (C=S). ^1^H-NMR spectrum of compound **16** exhibited singlet at 10.8 ppm. attributed to 2NH groups, which exchangeable with D_2_O. Mass spectrum of compound **16** exhibited a molecular ion peak m/z at of 358 (M^+^) (17.53) with a base peak appeared at 156 (100). ^13^C-NMR spectrum of compound **16** showed singlet at 178.6 ppm attributed to (C=S). IR spectrum of compound **17** revealed the characteristic bands at 3363, 3218, 3154 (NH, NH_2_), 3034 (CH arom.), 2943, 2836 (CH aliph.), 1590 (C=N), 1324, 1154 (SO_2_), 1241 (C=S). ^1^H-NMR spectrum of compound **17** exhibited singlets 10.1 and 13.8 ppm. attributed to 2NH groups, which exchangeable with D_2_O. Mass spectrum of compound **17** exhibited a molecular ion peak m/z at of 372 (M^+^) (21.22) with a base peak appeared at 141 (100). ^13^C-NMR spectrum of compound **17** showed singlet at 179.3 ppm attributed to (C=S).

### In-vitro anticancer evaluation

The synthesized compounds were evaluated for their in vitro anticancer activity against human lung cancer cell line (A549-Raw), cervical (Hela) cancer cell line, colorectal cell line (Lovo) and breast cancer cell line (MDA-MB231) using 2′7′dichlorofluorescein (DCF) and Doxorubicin as reference drugs in this study. The relationship between surviving fraction and drug concentration was plotted to obtain the survival curve of cancer cell lines. The response parameter calculated was the IC_50_ value, which corresponds to the concentration required for 50 % inhibition of cell viability. The results are presented in Table 1.

**Table 1:** In vitro anticancer screening of the newly synthesized compounds against four cancer cell lines

Compound no.	A549-Raw (lung cancer cells)	Hela cells	Lovo (colorectal cancer cells)	MDA-MB231 (breast cancer cells)
IC_50_ (µg ml^−1^)				
**2**	99.59	NA	148.33	NA
**3**	29.12	35.63	39.83	26.28
**4**	NA	NA	NA	NA
**5**	NA	NA	NA	NA
**6**	87.72	80.65	NA	64.56
**7**	NA	NA	NA	NA
**8**	82.25	78.47	91.17	69.04
**9**	55.08	42.16	66.27	38.46
**10**	85.11	85.11	98.24	39.13
**11**	NA	NA	NA	NA
**12**	NA	NA	NA	NA
**13**	NA	NA	NA	NA
**14**	NA	NA	NA	NA
**15**	74.75	93.42	NA	53.06
**16**	114.28	NA	NA	64.71
**17**	NA	NA	NA	NA
**DCF**	124.87	54.07	114.12	113.94
**Doxorubicin**	164.46	70.01	217.15	15.41

Regarding the cytotoxic activity on lung cancer cell line (A549), compounds **2**, **3**, **6**, **8**, **9**, **15** and **16** were active with IC_50_ ranging between 29.12 and 114.28 µg ml^−1^. The most active compound was the *n*-heptane thiourea derivative **3**. In case of cervical cancer cell line (Hela), compounds **3**, **6**, **8**, **9**, **10** and **15** were active with IC_50_ ranging between 35.63 and 93.42 µg ml^−1^. The most active compounds was again the *n*-hepatne thiourea derivative **3**.

For the colorectal cell line (Lovo), compounds **2**, **3**, **8**, **9** and **10** were active with IC_50_ ranging between 39.83 and 148.33 µg ml^−1^ and once again the most active compound was *n*-hepatne thiourea derivative **3**. Finally, the activity on breast cancer cell line (MDA-MB231) was exhibited by compounds **3**, **6**, **8**, **9**, **10**, **15** and **16** with IC_50_ ranging between 26.28 and 69.04 µg ml^−1^ with less activity than Doxorubicin. The same compound (*n*-hepatne thiourea derivative **3**) was the most active compound.

### Structure activity relationship

In a closer look to the biological results we can see that: the thiourea derivatives **3**, **6**, **8**, **9**, **10**, **15** and **16** were the active compounds on most of the cell lines while the rest of the compounds were inactive. It was obvious that incorporating an *n*-heptane aliphatic substitution as in compound **3** gave the most activity on all cell line. This activity was reduced upon replacing this substituent with another tricyclic aliphatic one as in compound **9.** In case of aromatic substitution the activity was retained but markedly decreased as in the 2-methyl-6-nitrophenyl thiourea derivative **6**, the 3-benzo[*d*][1,3]dioxol-5-ylmethyl thiourea derivative **8**, the 3-(5,6-dimethylbenzo[d]thiazol-2-yl)thiourea derivative **10**, the tetrahydronaphthalen derivative **15** and the quinoline derivative **16**.

Comparing compound **3** which was the most active compound among the newly synthesized compounds with the reference drug Doxorubicin we can see that: compound **3** was more active that Doxorubicin as cytotoxic agents on lung cancer cell line, Hella cells and colorectal cancer cells with IC_50_ value of 29.12, 35.63 and 39.83 µg ml^−1^, respectively. However, in case of breast cancer cell line compound **3** was less active than Doxorubicin with IC_50_ value of 26.28 µg ml^−1^.

### Molecular docking

**Fig. 1:**
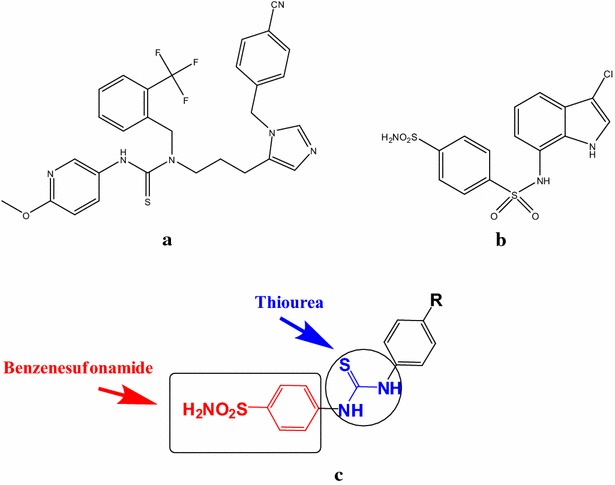
**a** YH345, **b** Indisulam, **c** general structure for the designed compounds

Mitogen-activated protein kinase-activated protein kinase 2 (MAPKAPK-2 or MK-2) is an important enzyme in signal transduction pathway controlling several pathways in cell proliferation [39]. MK-2 inhibition is one of the strategies of discovering new anticancer agents [40]. Recently, several urea and thiourea derivatives have shown good inhibitory activity on MK-2 [41]. Based on the thiourea scaffold of our newly synthesized compounds and as a trial to suggest a mechanism of action for their cytotoxic activity, molecular docking was performed on the active site of MK-2 for the most active compound. The protein data bank file (PDB:3WI6). The file contains MK-2 enzyme co-crystalized with an inhibitor. All docking procedures were achieved by MOE (Molecular Operating Environment) software 10.2008 provided by chemical computing group, Canada. The inhibitor interacts with MK-2 active site with four hydrogen bonds involving Glu 190, Leu 141, Asn 191 ans Asp 207 (Fig. 1). The docking protocol was validated by redocking of the ligand on the active site of MK-2 enzyme with energy score (S) = −15.4978 kcal mol^−1^ and root mean square deviation (RMSD) = 1.1457.

The active compounds were docked on the active site of MK-2 using the same docking protocol. Energy scores and amino acid interactions were displayed in Table 2.

**Table 2:** Binding scores and amino acid interactions of the docked compounds on the active site of mitogen activated kinase (MK-2)

Compound no.	S Kcal mol^−1^	Amino acid interactions	Interacting groups	Type of interaction	H bond length Å
**3**	−15.4739	Leu 141	NH_2_	H-bond (donor)	1.41
			SO_2_	H-bond (acceptor)	2.96
		Asp 207	NH	H-bond (donor)	2.16
**6**	−13.1926	Lys 188	SO_2_	H-bond (acceptor)	2.58
**8**	−17.6042	Lys 188	SO_2_	H-bond (acceptor)	2.56
**9**	−10.2371	Asp 207	NH_2_	H-bond (donor)	1.70
**10**	−18.9455	Glu 145	NH	H-bond (donor)	1.49
			NH	H-bond (donor)	1.85
		Lys 188	SO_2_	H-bond (acceptor)	3.02
**15**	−13.5639	Lys 188	SO_2_	H-bond (acceptor)	2.33
		Asp 207	NH	H-bond (donor)	2.22
**16**	−20.1443	Lys 188	SO_2_	H-bond (acceptor)	2.61

**Fig. 2:**
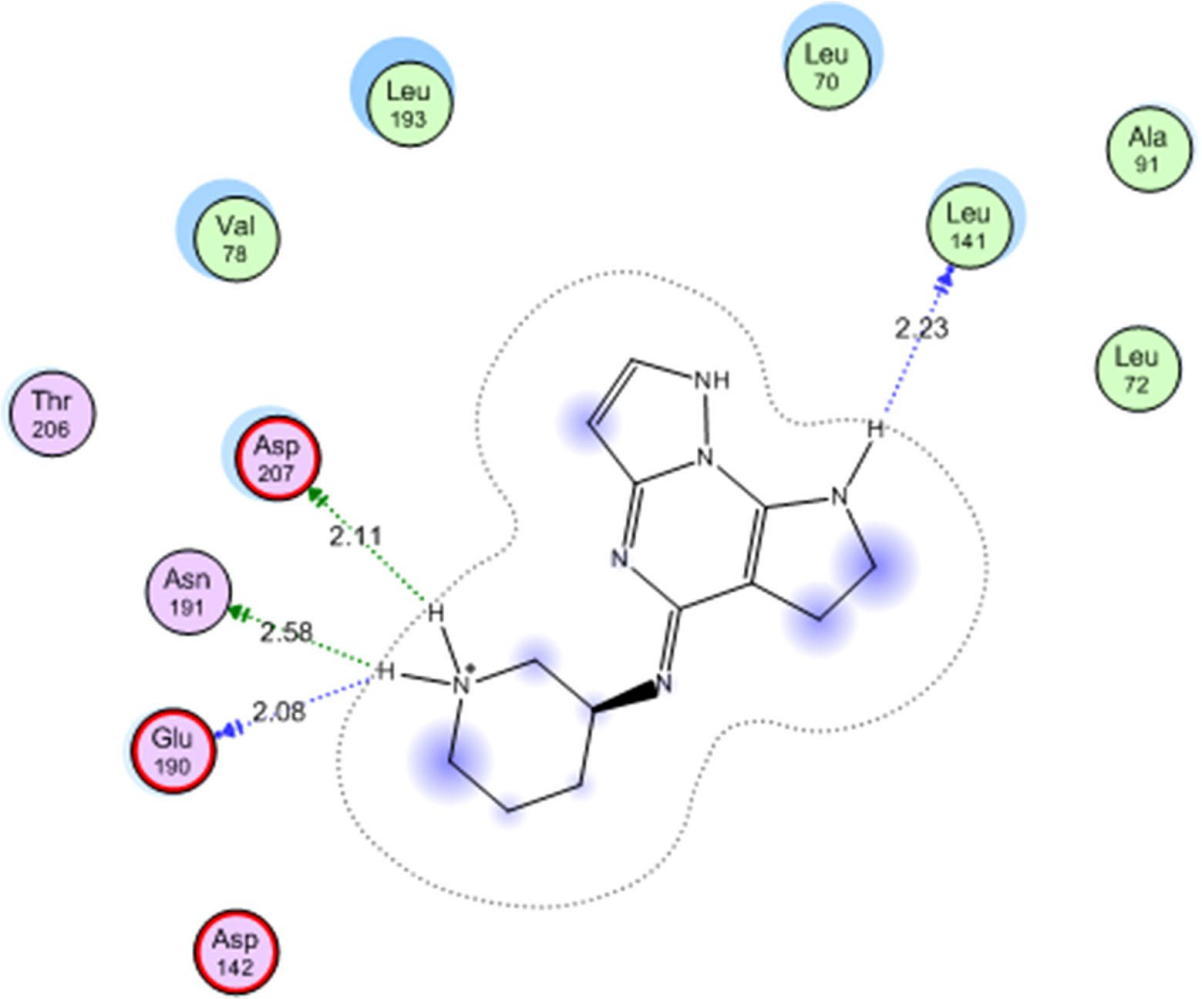
Co-crystalized lignd in the active site of mitogen activated kinase (MK-2)

**Fig. 3:**
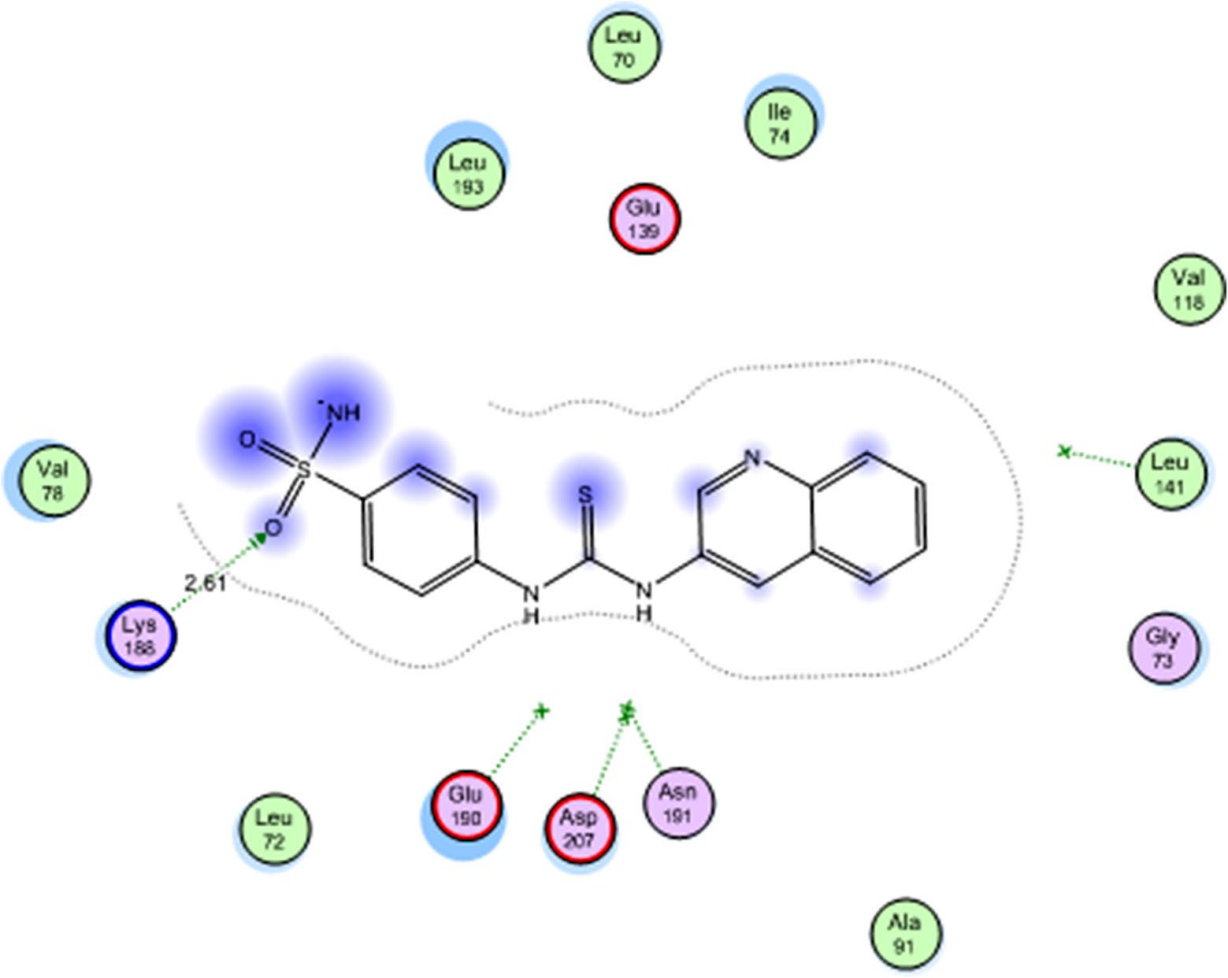
Compound **16** in the active site of mitogen activated kinase (MK-2)

**Fig. 4:**
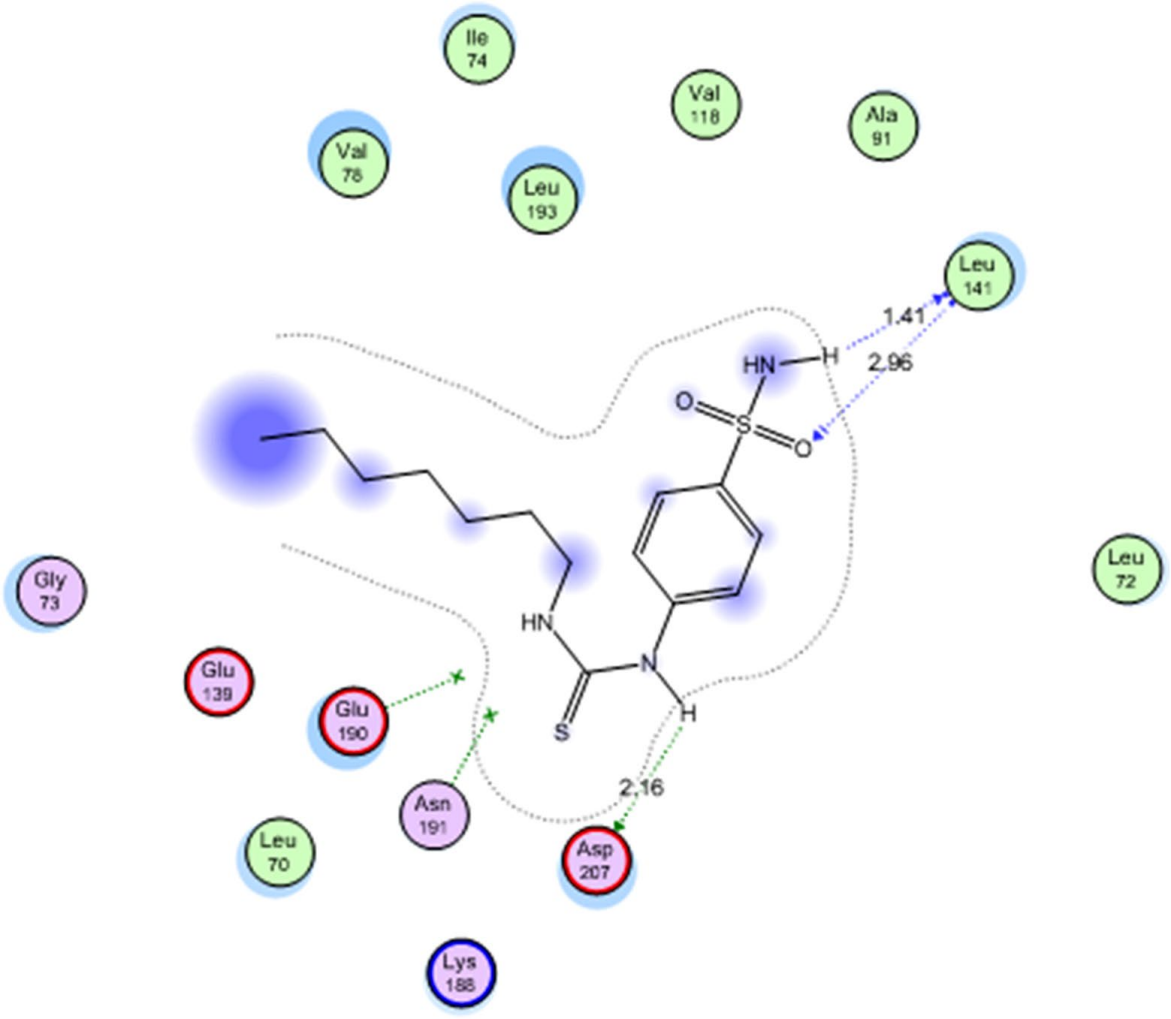
Compound **3** in the active site of mitogen activated kinase (MK-2)

All the docked compounds were fit on the active site of MK-2 with energy scores ranging between −10.2371 and −20.1443 kcal mol^−1^. Best docking score was exhibited by compound **16** which interacted with Lys 188 with one hydrogen bond (Fig. 2) while the best amino acid interaction was exhibited by compound **3** which interacted with Leu 141 by two hydrogen bonds and Asp 207 with one hydrogen bond (Figs. 3, 4). The same previous two amino acids interacted also with the co-crystalized inhibitor in a comparable manner.

## Experimental

### General chemistry

Melting points (uncorrected) were and determined in open capillary on a Gallen Kamp melting point apparatus (Sanyo Gallen Kamp, UK). Precoated silica gel plates *(Kieselgel* 0.25 mm, 60 F254, Merck, Germany) were used for thin layer chromatography. A developing solvent system of chloroform/methanol (8:2) was used and the spots were detected by ultraviolet light. IR spectra (KBr disc) were recorded using an FT-IR spectrophotometer (Perkin Elmer, USA). ^1^H-NMR spectra were scanned on an NMR spectrophotometer (Bruker AXS Inc., Switzerland), operating at 500 MHz for ^1^H- and 125.76 MHz for ^13^C. Chemical shifts are expressed in δ-values (ppm) relative to TMS as an internal standard, using DMSO-*d*_*6*_ as a solvent. Elemental analyses were done on a model 2400 CHNSO analyser (Perkin Elmer, USA). All the values were within ±0.4 % of the theoretical values. All reagents used were of AR grads.

### Synthesis of thioureidobenzenesulfonamide derivatives (**3**–**17**)

#### General procedure

A mixture of 4-isothiocyanatobenzenesulfonamide **2** (2.14 g, 0.01 mol) and amines (0.012 mol) in dry dimethylformamide (15 ml) containing three drops of triethylamine was refluxed for 24 h, then left to cool. The solid product formed upon pouring onto ice/water was collected by filtration and recrystallized from ethanol–dimethylformamide to give **3**–**17**, respectively.

#### 4-(3-Heptylthioureido)benzenesulfonamide (**3**)

Yield, 92 %; m.p. 124.7 °C. IR (KBr, cm^−1^): 3218, 3143 (NH, NH_2_), 3087 (CH arom.), 2926, 2853 (CH aliph.), 1376, 1150 (SO_2_), 1254 (C=S). ^1^H-NMR (DMSO-d_2_): 0.8 [t, 2H, CH_3_], 1.2–1.4 [m, 10H, 5CH_2_], 3.3 [m, 2H, NHCH_2_], 7.3–7.9 [m, 6H, Ar–H + SO_2_NH_2_], 9.3, 10.4 [2 s, 2NH, exchangeable with D_2_O]. ^13^C-NMR (DMSO-d_6_): 14.2, 22.4, 26.2, 28.6, 29.0, 31.5, 43.9, 119.4 (2), 127.4 (2), 134.7, 143.0, 177.4.MS m/z (%): 329 (M^+^) (14.41), 155 (100). Anal.Calcd. For C_14_H_23_N_3_O_2_S_2_ (329): C, 51.03; H, 7.04; N, 12.75. Found: C, 51.29; H, 6.79; N, 12.45.

#### 4-(3-(4-Hydroxyphenyl)thioureido)benzenesulfonamide (**4**)

Yield, 88 %; m.p.192.9 °C. IR (KBr, cm^−1^): 3363 (OH), 3280, 3143 (NH, NH_2_), 3090 (CH arom.), 1393, 1182 (SO_2_), 1274 (C=S).^1^H-NMR (DMSO-d_2_): 6.7–7.9 [m, 10H, Ar–H + SO_2_NH_2_], 10.2, 11.4, [2 s, 2H, 2NH, exchangeable with D_2_O], 13.1 [s, 1H, OH, exchangeable with D_2_O], ^13^C-NMR (DMSO-d_6_): 112.9 (2), 122.8 (2), 126.7 (2), 127.1 (2), 127.9, 139.8, 140.3, 157.6, 180.1. MS m/z (%): 323 (M^+^) (9.03), 91 (100). Anal.Calcd. For C_13_H_13_N_3_O_3_S_2_ (323): C, 48.28; H, 4.05; N, 12.99. Found: C, 48.55; H, 4.31; N, 13.29.

#### 4-(3-(3,5-Dimethoxyphenyl)thioureido)benzenesulfonamide (**5**)

Yield, 77 %; m.p. 160.3 °C. IR (KBr, cm^−1^): 3317, 3254, 3173 (NH, NH_2_), 3100 (CH arom.), 2963, 2938, 2829 (CH aliph.), 1363, 1156 (SO_2_), 1259 (C=S). ^1^H-NMR (DMSO-d_2_): 3.9 [s, 6H, 2OCH_3_], 6.3–7.8 [m, 8H, Ar–H + SO_2_NH_2_], 9.8 [s, 2H, 2NH, exchangeable with D_2_O].^13^C-NMR (DMSO-d_6_): 56.1 (2), 96.8, 102.0 (2), 123.2 (2), 126.6 (2), 141.4 (2), 143.1, 160.6 (2), 179.3. MS m/z (%): 367 (M^+^) (17.8), 76 (100). Anal.Calcd. For C_15_H_17_N_3_O_4_S_2_ (367): C, 49.03; H, 4. 66; N, 11.44. Found: C, 48.74; H, 4.29; N, 11.17.

#### 4-(3-(2-Methyl-6-nitrophenyl)thioureido)benzenesulfonamide (**6**)

Yield, 81 %; m.p. 226.0 °C. IR (KBr, cm^−1^): 3353, 3243, 3171 (NH, NH_2_), 3009 (CH arom.), 1340, 1161 (SO_2_), 1290 (C=S).^1^H-NMR (DMSO-d_2_): 2.2 [s, 3H, CH_3_], 6.5–7.8 [m, 9H, Ar–H + SO_2_NH_2_], 10.3 [s, 2H, 2NH exchangeable with D_2_O]. ^13^C-NMR (DMSO-d_6_): 18.3, 123.3, 123.9 (2), 126.7, 126.8, 127.8 (2), 131.3, 136.5 (2), 139.8, 142.8, 180.1. MS m/z (%): 366 (M^+^) (15.8), 133 (100). Anal.Calcd. For C_14_H_14_N_4_O_4_S_2_ (366): C, 45.89; H, 3.85; N, 15.29. Found: C, 45.57; H, 3.54; N, 15.61.

#### 4-(3-Benzo[*d*][1,3]dioxol-5-ylthioureido)benzenesulfonamide (**7**)

Yield, 86 %; m.p. 136.6 °C. IR (KBr, cm^−1^): 3325, 3241 (NH, NH_2_), 3100 (CH arom.), 1331, 1156 (SO_2_), 1241 (C=S). ^1^ H-NMR (DMSO-d_2_): 6.0 [s, 2H, CH_2_], 6.7–7.9 [m, 9H, Ar–H + SO_2_NH_2_], 9.5 [s, 2H, 2NH, exchangeable with D_2_O]. ^13^C-NMR (DMSO-d_6_): 101.2, 107.1, 109.1, 117.8, 123.1 (2), 126.5 (2), 133.4, 134.6, 142.4, 143.2, 147.3, 180.6. MS m/z (%): 351 (M^+^) (34.64), 93 (100). Anal.Calcd. For C_14_H_13_N_3_O_4_S_2_ (351): C, 47.85; H, 3.73; N, 11.96. Found: C, 47.49; H, 3.43; N, 11.62.

#### 4-(3-Benzo[*d*][1,3]dioxol-5-ylmethyl)thioureido)benzenesulfonamide (**8**)

Yield, 68 %; m.p. 140.8 °C. IR (KBr, cm^−1^): 3384, 3348, 3206 (NH, NH_2_), 3003 (CH arom.), 1377, 1185 (SO_2_), 1294 (C=S).^1^H-NMR (DMSO-d_2_): 4.3 [s, 2H, CH_2_], 6.0 [s, 2H, OCH_2_O], 6.7–7.7 [m, 9H, Ar–H + SO_2_NH_2_], 7.8 [s, 2H, +2NH, exchangeable with D_2_O]. ^13^C-NMR (DMSO-d_6_): 63.8, 101.2, 106.4, 108.2, 120.8, 121.4 (2), 131.2 (2), 133.5, 134.0, 146.4, 147.6, 148.3, 161.1. MS m/z (%): 365 (M^+^) (18.42), 135 (100). Anal.Calcd. For C_15_H_15_N_3_O_4_S_2_ (365): C, 49.30; H, 4.14; N, 11.50. Found: C, 49.05; H, 4.46; N, 11.19.

#### 4-(3-(1-Adamanylamine)thioureidobenzenesulfonamide (**9**)

Yield, 80 %; m.p. 174.5 °C. IR (KBr, cm^−1^): 3434, 3354 (NH, NH_2_), 3100 (CH arom.), 2997, 2906, 2851 (CH aliph.), 1396, 1186 (SO_2_), 1282 (C=S).^1^H-NMR (DMSO-d_2_): 1.6–1.9 [m, 12H, 6CH_2_], 2.2–2.4 [m, 3H, 3 CH], 6.9–7.9 [m, 6H, Ar–H + SO_2_NH_2_], 11.4 [s, 2H, 2NH, exchangeable with D_2_O].^13^C-NMR (DMSO-d_6_): 28.8 (3), 35.3 (3), 40.5 (3), 44.9, 126.4 (2), 129.1 (2), 131.8, 142.7, 179.9. MS m/z (%): 366 (M^+^) (9.32), 154 (100). Anal.Calcd. For C_17_H_23_N_3_O_2_S_2_ (366): C, 55.86; H, 6.34; N, 11.50. Found: C, 55.50; H, 6.68; N, 11.18.

#### 4-(3-(5,6-Dimethylbenzo[d]thiazol-2-yl)thioureido)benzenesulfonamide (**10**)

Yield, 84 %; m.p. 252.1 °C. IR (KBr, cm^−1^): 3359, 3257, 3143 (NH, NH_2_), 3031 (CH arom.), 2954, 2851 (CH aliph.), 1594 (C=N), 1381, 1186 (SO_2_), 1296 (C=S).^1^H-NMR (DMSO-d_2_): 2.2 [s, 6H, 2CH_3_], 7.2–8.0 [m, 8H, Ar–H + SO_2_NH_2_], 10.2, 13.0 [2 s, 2H, 2NH, exchangeable with D_2_O].^13^C-NMR (DMSO-d_6_): 19.9, 20.4, 118.5, 121.5, 123.3 (2), 126.6, 127.1, 127.8 (2), 133.0, 136.2, 139.8, 143.1, 151.9, 180.0 (2).MS m/z (%): 393 (M^+^) (16.9), 162 (100). Anal.Calcd. For C_16_H_16_N_4_O_2_S_3_ (393): C, 48.96; H, 4.11; N, 14.27. Found: C, 48.66; H, 3.85; N, 14.54.

#### 4-(3-(6-Ethoxybenzo[d]thiazol-2-yl)thioureido)benzenesulfonamide (**11**)

Yield, 78 %;m.p. 153.6 °C. IR (KBr, cm^−1^): 3410, 3334, 3195 (NH, NH_2_), 3069 (CH arom.), 2974, 2925, 2843 (CH aliph.), 1595 (C=N), 1393, 1123 (SO_2_), 1256 (C=S). ^1^H-NMR (DMSO-d_2_): 1.3 [t, 3H, CH_3_], 4.0 [q, 2H, CH_2_], 6.9–8.0 [m, 9H, Ar–H + SO_2_NH_2_], 10.3, 11.2 [2 s, 2H, 2NH, exchangeable with D_2_O]. ^13^C-NMR (DMSO-d_6_): 15.0, 66.8, 106.0, 115.3, 118.2, 120.4 (2), 127.7 (2), 132.7, 138.2, 140.9, 142.1, 157.7, 177.1, 180.1. MS m/z (%): 409 (M^+^) (1.85), 156 (100). Anal.Calcd. For C_16_H_16_N_4_O_3_S_3_ (409): C, 47.04; H, 3.95;N, 13.71. Found: C, 47.34; H, 3.67; N, 13.39.

#### 4-(3-(6-Nitrobenzo[d]thiazol-2-yl))thioureido)benzenesulfonamide (**12**)

Yield, 65 %; m.p. 205.8 °C. IR (KBr, cm^−1^): 3384, 3261, 3165 (NH, NH_2_), 3097 (CH arom.), 1595 (C=N), 1331, 1185 (SO_2_), 1252 (C=S).^1^H-NMR (DMSO-d_2_): 7.1–8.9 [m, 9H, Ar–H + SO_2_NH_2_], 10.5, 12.0 [2 s, 2H, 2NH, exchangeable with D_2_O]. ^13^C-NMR (DMSO-d_6_): 119.6 (2), 123.1 (3), 126.6 (3), 139.8 (2), 142.8, 161.2179.9 (2). MS m/z (%): 409 (M^+^) (13.43), 178 (100). Anal.Calcd. For C_14_H_11_N_5_O_4_S_3_ (409): C, 41.07; H, 2.71; N, 17.10. Found: C, 41.31; H, 2.40; N, 17.43.

#### 4-(3-(5-(Bromopyridin-2-yl)thioureido)benzenesulfonamide (**13**)

Yield, 72 %; m.p. 247.0 °C. IR (KBr, cm^−1^): 3326, 3175 (NH, NH_2_), 3088 (CH arom.), 1572 (C=N), 1356, 1192 (SO_2_), 1211 (C=S). ^1^H-NMR (DMSO-d_2_): 6.8–8.3 [m, 8H, Ar–H + SO_2_NH_2_], 12.4 [s, 2H, 2NH, exchangeable with D_2_O]. ^13^C-NMR (DMSO-d_6_): 105.5, 124.8 (2), 128.9 (2), 134.6, 140.8, 158.5, 159.3 (2), 178.6.MS m/z (%): 388 (M^+^) (11.81), 157 (100). Anal.Calcd. For C_11_H_10_BrN_5_O_2_S_2_ (388): C, 34.03; H, 2.60; N, 18.04. Found: C, 34.28; H, 2.27; N, 18.37.

#### 4-(3-Pyrazin-2-ylthioureido)benzenesulfonamide (**14**)

Yield, 80 %; m.p. 185.3 °C. IR (KBr, cm^−1^): 3378, 3240, 3155 (NH, NH_2_), 3100 (CH arom.), 1601 (C=N), 1346, 1199 (SO_2_), 1270 (C=S).^1^H-NMR (DMSO-d_2_): 7.2–8.7 [m, 9H, Ar–H + SO_2_NH_2_], 11.3, 13.0 [2 s, 2H, 2NH, exchangeable with D_2_O]. ^13^C-NMR (DMSO-d_6_): 123.1 (2), 126.7 (2), 137.1, 138.4, 138.5, 139.9, 140.3, 149.7, 179.0. MS m/z (%): 309 (M^+^) (12.83), 79 (100). Anal.Calcd. For C_11_H_11_N_5_O_2_S_2_ (309): C, 42.71; H, 3.58; N, 22.64. Found: C, 42.38; H, 3.84; N, 22.29.

#### 4-(3-(5,6,7,8-Tetrahydronaphthalen-1-yl)thioureido)benzenesulfonamide (**15**)

Yield, 76 %; m.p. 171.8 °C. IR (KBr, cm^−1^): 3413, 3354, 3152 (NH, NH_2_), 3083 (CH arom.), 2982, 2935, 2831 (CH aliph.), 1351, 1159 (SO_2_), 1264 (C=S).^1^H-NMR (DMSO-d_2_): 1.8–2.8 [m, 8H, 4CH_2_, cyclo], 7.0–8.0 [m, 9H, Ar–H + SO_2_NH_2_], 9.0[s, 2H, 2NH, exchangeable with D_2_O]. ^13^C-NMR (DMSO-d_6_): 22.7 (2), 24.8, 29.6, 117.4, 120.8 (2), 124.0, 125.7, 127.4 (2), 134.5, 137.1, 137.4, 137.6, 146.4, 181.5.MS m/z (%): 361 (M^+^) (26.34), 177 (100). Anal.Calcd. For C_17_H_19_N_3_O_2_S_2_ (361): C, 56.48; H, 5.30; N, 11.62. Found: C, 56.12; H, 5.03; N, 11.36.

#### 4-(3-Quinolin-3-ylthioureido)benzenesulfonamide (**16**)

Yield, 66 %; m.p. 214.6 °C. IR (KBr, cm^−1^): 3373, 3246, 3164 (NH, NH_2_), 3077 (CH arom.), 1595 (C=N), 1365, 1150 (SO_2_), 1293 (C=S). ^1^H-NMR (DMSO-d_2_): 6.8–8.5 [m, 12H, Ar–H + SO_2_NH_2_], 10.8 [s, 2H, 2NH, exchangeable with D_2_O].^13^C-NMR (DMSO-d_6_): 127.3, 127.7, 128.6, 129.1 (2), 130.0 (2), 132.0, 134.3 (2), 137.9 (2), 142.8, 178.6. MS m/z (%): 358 (M^+^) (17.53), 156 (100). Anal.Calcd. For C_16_H_14_N_4_O_2_S_2_ (358): C, 53.61; H, 3.94; N, 15.63. Found: C, 53.36; H, 3.62; N, 15.36.

#### 4-(3-(2-Methylquinolin-4-yl)thioureido)benzenesulfonamide (**17**)

Yield, 71 %; m.p. 192.3 °C. IR (KBr, cm^−1^): 3363, 3218, 3154 (NH, NH_2_), 3034 (CH arom.), 2943, 2836 (CH aliph.), 1590 (C=N), 1324, 1154 (SO_2_), 1241 (C=S). ^1^H-NMR (DMSO-d_2_): 2.6 [s, 3H, CH_3_], 6.6–8.8 [m, 11H, Ar–H + SO_2_NH_2_], 10.1, 13.8[2 s, 2H, 2NH, exchangeable with D_2_O]. ^13^C-NMR (DMSO-d_6_): 19.9, 102.0, 108.3, 121.1 (2), 122.9, 124.0, 126.1, 127.8, 128.0 (2), 137.3, 139.5, 143.1, 151.7, 158.0, 179.3. MS m/z (%): 372 (M^+^) (21.22), 141 (100). Anal.Calcd. For C_17_H_16_N_4_O_2_S_2_ (372): C, 54.82; H, 4.33; N, 15.04. Found: C, 54.51; H, 4.09; N, 15.31.

### In vitro anticancer evaluation

#### Cell culture

Human cancer cell lines HeLa (cervical), A549 (lungs) and Lovo (colorectal) were grown in DMEM + GlutaMax (Invitrogen), and MDA MB321 (breast) were grown in DMEM-F12 + GlutaMax) medium (invitrogen), supplemented with 10 % heat-inactivated bovine serum (Gibco) and 1× penicillin–streptomycin (Gibco) at 37 °C in a humified chamber with 5 % CO_2_ supply.

#### Cytotoxicity assay

The in vitro anticancer screening was done at pharmacognosy Department, College of Pharmacy, King Saud University, Riyadh, Saudi Arabia. Cells were seeded (10^5^ cells/well) in 96-well flat-bottom plates (Becton–Dickinson Labware) a day before treatment and grown overnight. Compounds were dissolved in dimethyl sulfoxide (DMSO; Sigma) and finally prepared as 1.0 mg ml^−1^ stocks, respectively in the culture media. The final concentration of DMSO never exceeded 0.1 % in the treatment doses. Four different doses of compounds (50, 25, 12.5 and 6.25 µg ml^−1^) were further prepared by diluting the stocks in culture media, and cells were treated (in triplicate/dose). 2′7′ dichlorofluorescein (DCF) was included as standard reference drug (positive control) and untreated culture was considered as negative control. The treated cultures were further incubated for 48 h. At 48 h post-treatment, cell viability test was performed using TACS MTT Cell Proliferation and Viability Assay Kit (TACS) as per manufacturer’s instructions. The optical density (OD) was recorded at 570 nm in a microplate reader (BioTek, ELx800) and cell survival fraction was determined. The cell survival fraction was calculated as [(A − B)/A], where A and B are the OD of untreated and of treated cells, respectively [42]. The IC_50_ values of the tested compound were estimated using the best fit regression curve method in Excel.

#### Microscopy

A direct visual investigation was made under an inverted microscope (Optica, 40× and 100×) to observe any morphological changes in the cells cultured with different treatment doses at 24 and 48 h.

#### Molecular docking

All the molecular modeling studies were carried out on an Intel Pentium 1.6 GHz processor, 512 MB memory with Windows XP operating system using Molecular Operating Environment (MOE, 10.2008) software. All the minimizations were performed with MOE until a RMSD gradient of 0.05 kcal mol^−1^ Å with MMFF94X force field and the partial charges were automatically calculated. The protein data bank file (PDB: 3WI6) was selected for this purpose. The file contains MK-2 enzyme co-crystalized with a ligand obtained from protein data bank. The enzyme was prepared for docking studies where: (1) Ligand molecule was removed from the enzyme active site. (2) Hydrogen atoms were added to the structure with their standard geometry. (3) MOE Alpha Site Finder was used for the active sites search in the enzyme structure and dummy atoms were created from the obtained alpha spheres. (4). The obtained model was then used in predicting the ligand enzymes interactions at the active site.

## Conclusions

In summary, we had synthesized a novel series of sulfonamide thiourea derivatives. Seven compounds **3**, **6**, **8**, **9**, **10**, **15** and **16** showed good anticancer activity against lung (A594 Raw), Hela, and Colorectal (Lovo) cancer cell lines with better or comparable activity to DCF. Moreover, molecular docking for these active compounds showed proper fitting on the active site of MK-2 enzyme suggesting their action as inhibitors for this enzyme but more investigation should be carried out in the future to explore precisely the mechanism of the action of the synthesized derivatives.
